# Simultaneous Presence of Bacteriochlorophyll and Xanthorhodopsin Genes in a Freshwater Bacterium

**DOI:** 10.1128/mSystems.01044-20

**Published:** 2020-12-22

**Authors:** Karel Kopejtka, Jürgen Tomasch, Yonghui Zeng, Vadim Selyanin, Marko Dachev, Kasia Piwosz, Martin Tichý, David Bína, Zdenko Gardian, Boyke Bunk, Henner Brinkmann, Robert Geffers, Ruben Sommaruga, Michal Koblížek

**Affiliations:** a Center Algatech, Institute of Microbiology of the Czech Academy of Science, Třeboň, Czechia; b Research Group Microbial Communication, Technical University of Braunschweig, Braunschweig, Germany; c Department of Environmental Science, Aarhus University, Aarhus, Denmark; d Institute of Plant Molecular Biology, Biology Center of the Czech Academy of Sciences, České Budějovice, Czechia; e Institute of Parasitology, Biology Center of the Czech Academy of Sciences, České Budějovice, Czechia; f Faculty of Science, University of South Bohemia, České Budějovice, Czechia; g Leibniz Institute DSMZ-German Collection of Microorganisms and Cell Cultures, Braunschweig, Germany; h Research Group Genome Analytics, Helmholtz Centre for Infection Research, Braunschweig, Germany; i Laboratory of Aquatic Photobiology and Plankton Ecology, Department of Ecology, University of Innsbruck, Innsbruck, Austria; University of Pretoria

**Keywords:** aerobic anoxygenic phototrophic bacteria, bacteriochlorophyll *a*, gene expression, photosynthesis gene cluster, rhodopsin, *Sphingomonadaceae*

## Abstract

Photoheterotrophic bacteria represent an important part of aquatic microbial communities. There exist two fundamentally different light-harvesting systems: bacteriochlorophyll-containing reaction centers or rhodopsins. Here, we report a photoheterotrophic *Sphingomonas* strain isolated from an oligotrophic lake, which contains complete sets of genes for both rhodopsin-based and bacteriochlorophyll-based phototrophy. Interestingly, the identified genes were not expressed when cultured in liquid organic media. Using reverse transcription quantitative PCR (RT-qPCR), RNA sequencing, and bacteriochlorophyll *a* quantification, we document that bacteriochlorophyll synthesis was repressed by high concentrations of glucose or galactose in the medium. Coactivation of photosynthesis genes together with genes for TonB-dependent transporters suggests the utilization of light energy for nutrient import. The photosynthetic units were formed by ring-shaped light-harvesting complex 1 and reaction centers with bacteriochlorophyll *a* and spirilloxanthin as the main light-harvesting pigments. The identified rhodopsin gene belonged to the xanthorhodopsin family, but it lacks salinixanthin antenna. In contrast to bacteriochlorophyll, the expression of xanthorhodopsin remained minimal under all experimental conditions tested. Since the gene was found in the same operon as a histidine kinase, we propose that it might serve as a light sensor. Our results document that photoheterotrophic *Sphingomonas* bacteria use the energy of light under carbon-limited conditions, while under carbon-replete conditions, they cover all their metabolic needs through oxidative phosphorylation.

**IMPORTANCE** Phototrophic organisms are key components of many natural environments. There exist two main phototrophic groups: species that collect light energy using various kinds of (bacterio)chlorophylls and species that utilize rhodopsins. Here, we present a freshwater bacterium *Sphingomonas* sp. strain AAP5 which contains genes for both light-harvesting systems. We show that bacteriochlorophyll-based reaction centers are repressed by light and/or glucose. On the other hand, the rhodopsin gene was not expressed significantly under any of the experimental conditions. This may indicate that rhodopsin in *Sphingomonas* may have other functions not linked to bioenergetics.

## Abstract (precis)

Phototrophic organisms are key components of many natural environments. There exist two main phototrophic groups: species that collect light energy using various kinds of (bacterio)chlorophylls and species that utilize rhodopsins.

## INTRODUCTION

The ability to use light energy is an important trait widespread within aquatic microbial communities. Photoautotrophic phytoplankton harvest light using chlorophyll, evolve oxygen, and fix inorganic carbon using RubisCO (1). In addition to these dominant oxygenic phototrophs, there exist a large number of photoheterotrophic organisms, which harvest light to supplement their mostly heterotrophic metabolism. There are two main groups of aquatic photoheterotrophic bacteria: aerobic anoxygenic phototrophic (AAP) bacteria and rhodopsin-containing bacteria. Both groups are commonly retrieved from euphotic zones of the world oceans (2–5) and from limnic environments (6, 7).

AAP bacteria harvest light using bacteriochlorophyll (BChl), but in contrast to purple nonsulfur photosynthetic bacteria, they are obligate aerobes requiring oxygen for their metabolism and growth (8). Upon illumination, they drive electron transport and pump protons across the membrane, which are subsequently utilized for ATP synthesis. The metabolic utilization of harvested energy has been demonstrated under laboratory conditions (9, 10) and in field experiments (11).

Rhodopsins represent a diverse family of molecules that serve multiple functions. While bacteriorhodopsins, proteorhodopsins (PR), and xanthorhodopsins (XR) serve as proton membrane pumps in *Proteobacteria* (12), XR is a PR-like proton pump containing in addition to retinal another chromophore, salinixanthin, which serves as a light-harvesting antenna (13, 14). Sensory rhodopsins serve as photoreceptors in vertebrates, including humans. In contrast to bacteriorhodopsin containing *Archaea*, the role of proteorhodopsin in bacteria remains ambiguous. The first experiments showed no growth stimulation by light in Pelagibacter ubique strain HTCC1062 (15). In contrast, the illumination of *Dokdonia* sp. strain MED134 (*Bacteriodetes*) and *Vibrio* sp. strain AND4 (*Gammaproteobacteria*) enhanced growth and increased survival under starvation conditions, which indicates that PR provided energy for growth (16–18). The potential coexistence of two different phototrophic mechanisms in a single AAP bacterium was suggested for Fulvimarina pelagi (order *Rhizobiales*), whose genome sequence contains a XR gene as well as photosynthetic genes (19). Recently, a co-occurrence of the *pufM* gene, which encodes the M subunit of the bacterial reaction centers, and XR-like genes was found in three *Roseiflexus* (phylum *Chloroflexi*) genomes (20, 21). In *Cyanobacteria*, sensory rhodopsins were found to accompany chlorophyll-based photosynthetic machinery (22–25).

Bacteria of the genus *Sphingomonas* (*Alphaproteobacteria*) are common in many environments, such as soils, fresh waters, or phyllospheres (26–31). While most of the cultured *Sphingomonas* species are heterotrophs, there also exist species employing BChl-based reaction centers (32, 33) and species containing rhodopsin genes (29, 34, 35). Culture-independent studies documented that *Sphingomonas* with BChl genes are very common in freshwater photoheterotrophic communities (11, 36–38). Analysis of freshwater bacterioplankton in the oligotrophic alpine lake Gossenköllesee (Tyrolean Alps, Austria) revealed that phototrophic *Sphingomonas* dominates the local AAP community (30).

Since no AAP *Sphingomonas* has been characterized in the laboratory, we revisited the Gossenköllesee and cultured novel *Sphingomonas* species. We characterized their photosynthetic apparatus and its gene expression to better understand how these organisms use photosynthesis in their natural environment.

## RESULTS

### Strain isolation and sequencing.

The agar plates were inoculated with samples from the alpine lake Gossenköllesee (39) in September 2012. After 3 weeks of incubation, a yellow BChl *a*-containing colony was identified using infrared (IR) fluorescence screening.

**FIG 1:**
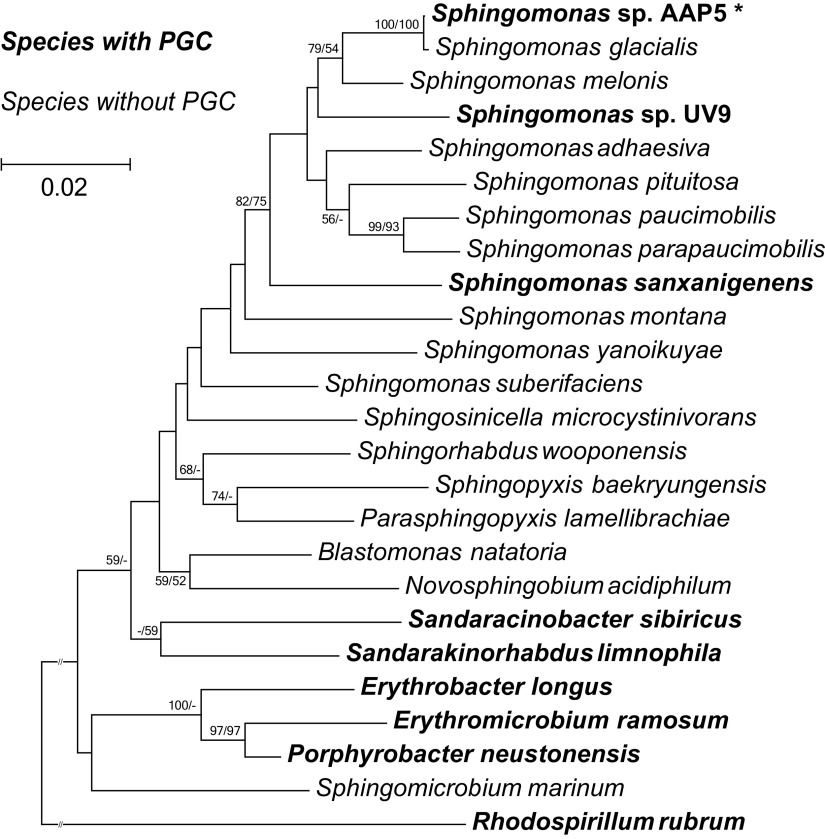
16S rRNA phylogenetic tree showing its position (marked with an asterisk) within the alpha-4 group of the *Proteobacteria*. The phylogenetic tree was based on 16S rRNA gene sequences downloaded from the SILVA database and NCBI GenBank (March 2019). Nucleotide sequences were aligned using ClustalW resulting in alignment with 1,302 conserved nucleotide positions (after ambiguously aligned regions and gaps were manually excluded). The phylogenetic tree was calculated using both neighbor-joining (NJ) and maximum likelihood (ML) algorithms and 1,500× bootstrap replicates. Rhodospirillum rubrum was used as an outgroup organism. The bar represents the number of changes per position. NJ/ML bootstrap values of >50% are shown. Species with PGC are shown in bold type.

The colony obtained was labeled AAP5, and its identity was inferred from its 16S rRNA gene. The constructed 16S rRNA phylogenetic tree (Fig. 1) showed that the AAP5 strain grouped with the genus *Sphingomonas* and formed a distinct cluster with S. glacialis (98.3% pairwise 16S rRNA sequence similarity) and S. melonis (96.5%).

The complete genome of AAP5 was sequenced by combining single-molecule real-time (SMRT) and Illumina technologies. The closed genome contained one circular chromosome and three plasmids, with a total length of 4.38 Mb encoding 4,128 genes. Genome characteristics are summarized in Table S2 in the supplemental material.

### Photosynthesis genes and regulators.

**FIG 2:**
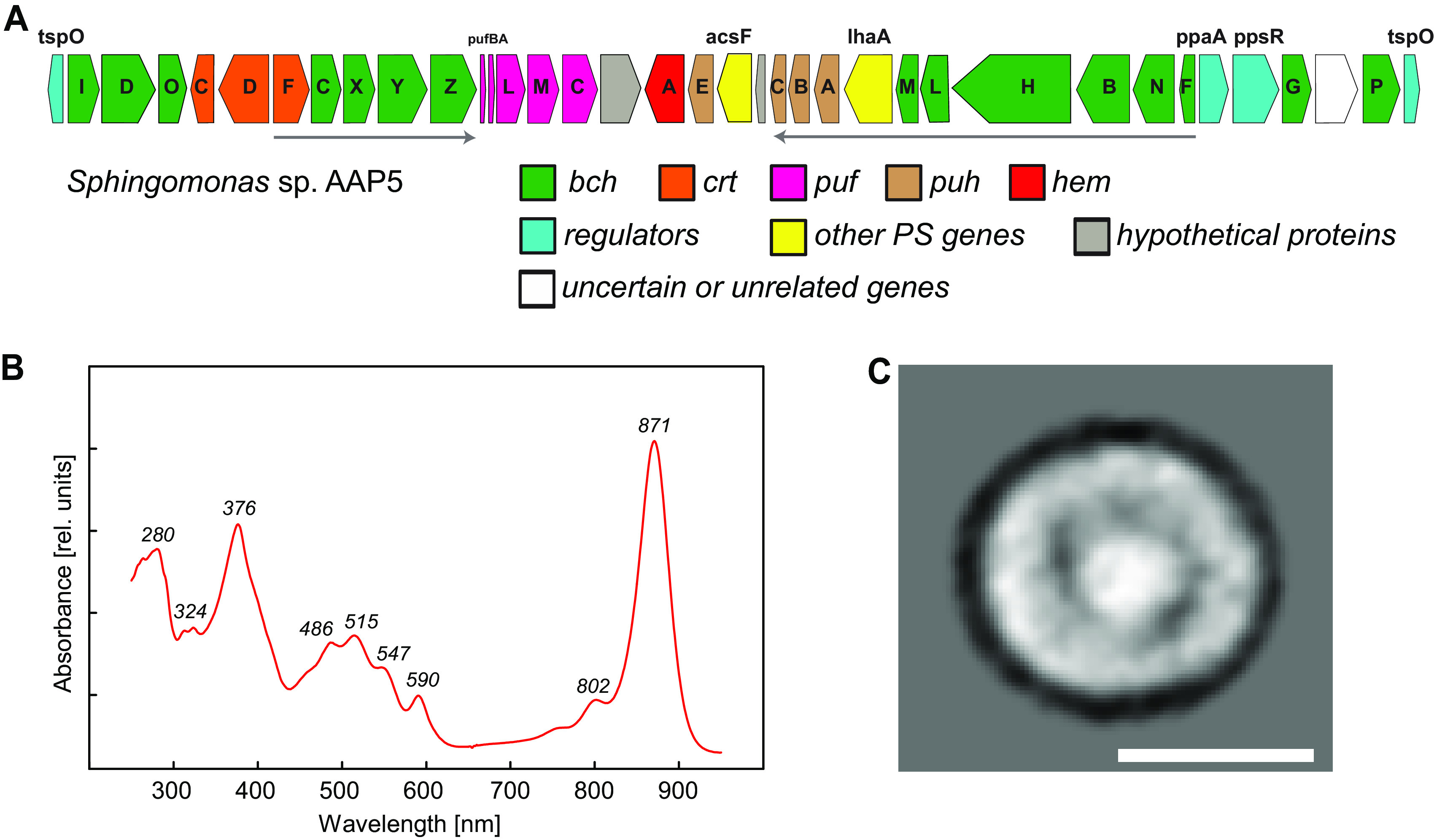
Photosynthetic competence of the AAP5 strain. (A) Gene organization of the photosynthesis gene cluster (PGC). *bch* (green), bacteriochlorophyll biosynthesis genes; *crt* (orange), carotenoid biosynthesis genes; *hem* (red), heme biosynthesis genes; *puf* (pink), genes encoding reaction center proteins; *puh* (brown), genes encoding reaction center assembly proteins; blue, regulatory genes; gray, hypothetical genes; white, uncertain or unrelated genes. Arrows show directions of superoperons *bchFNBHLM-lhaA-puhABC* and *crtF-bchCXYZ*. (B) Absorption spectrum of PS membranes. (C) Top view projection map of the PS complex. Bar, 10 nm. PS, photosynthetic.

The AAP5 genome contained one continuous 38.6-kb-long photosynthesis gene cluster (PGC) (Fig. 2A). The PGC encompasses the *puf* operon encoding type 2 photosynthetic reaction center (RC) subunits, and the complete set of genes for bacteriochlorophyll synthesis (*bch* genes, *acsF*). Only three genes for carotenoid biosynthesis were located inside the PGC (*crtF*, *crtD*, and *crtC*), while the remaining genes were located outside the PGC. The *puc* operon, encoding the peripheral light-harvesting complexes, was missing. The PGC contained the *hemA* gene (E2E30_16310) which seems to be a common feature of all AAP species in *Alphaproteobacteria* (40). Regulatory proteins were represented by PpaA (E2E30_16380) and PpsR (E2E30_16385). Interestingly, two open reading frames (ORFs) (E2E30_16220 and E2E30_16405) coding for the transcriptional modulator TspO were situated at opposite ends of the PGC. TspO is a membrane protein facilitating efflux of porphyrins and modulating PpsR activity in Rhodobacter sphaeroides (41). In Dinoroseobacter shibae, *tspO* is under the control of the singlet oxygen response regulator RpoE (42).

The synthesis of the photosynthetic (PS) apparatus is usually regulated in response to environmental conditions; therefore, we also searched for genes potentially involved in such regulation. The AAP5 genome contained 29 sensor histidine (His) kinases and 24 response regulators. Additionally, it harbors seven hybrid His kinase/response regulators, a putative bacteriophytochrome (E2E30_06640), and a BLUF domain-containing protein (E2E30_12925). Phytochromes are known to register red and far-red light (43), whereas BLUF domain has been shown to detect blue light (44).

### Photosynthetic complexes.

To purify and characterize BChl *a*-containing PS complexes, cells were harvested from IR-positive agar plates. High-performance liquid chromatography (HPLC) pigment analysis identified nostoxanthin as the main carotenoid (see Fig. S1 in the supplemental material). During purification, all nostoxanthin was removed, suggesting that it was not bound to the PS complexes, and thus, it does not have any light-harvesting function. No XR was found in the membrane fraction during purification. The absorption spectrum of the purified complex resembled very closely that of Rhodospirillum rubrum (45). It had three carotenoid absorption bands at 486, 515, and 547 nm and one near-infrared (NIR) absorption band at 871 nm (Fig. 2B) indicating the presence of light-harvesting complex 1 (LH1). The purified PS complexes were further investigated by electron microscopy. The single-particle analysis of the images obtained revealed circular particles with an outer diameter of ∼12 nm and an area of higher density in the center. This represents regular LH1-RC complexes composed of a single ring of light-harvesting LH1 complexes surrounding the reaction center (Fig. 2C). The main carotenoid present in the PS complexes was spirilloxanthin (Fig. S1). Aside from the main BChl *a* form, its phytyl derivative, there was also its geranylgeranyl, H_2_-geranylgeranyl and H_4_-geranylgeranyl derivative.

10.1128/mSystems.01044-20.1 FIG S1 Reversed-phase chromatography of AAP5 pigments. Carotenoids were monitored at 470 nm and bacteriochlorophyll *a* (Bchl *a*) at 770 nm. Note that traces are shifted vertically. Numbers above peaks indicate main pigments: 1, nostoxanthin; 2, BChl *a*_GG_ (geranylgeranyl); 3, BChl *a*_GGH2_ (dihydrogeranylgeranyl); 4, BChl *a*_GGH4_ (tetrahydrogeranylgeranyl); 5, BChl *a*_P_ (phytol); 6, spirilloxanthin. Download FIG S1, EPS file, 1.6 MB. Copyright © 2020 Kopejtka et al. 2020 Kopejtka et al. This content is distributed under the terms of the Creative Commons Attribution 4.0 International license.

### Rhodopsin and genes for retinal biosynthesis.

**FIG 3:**
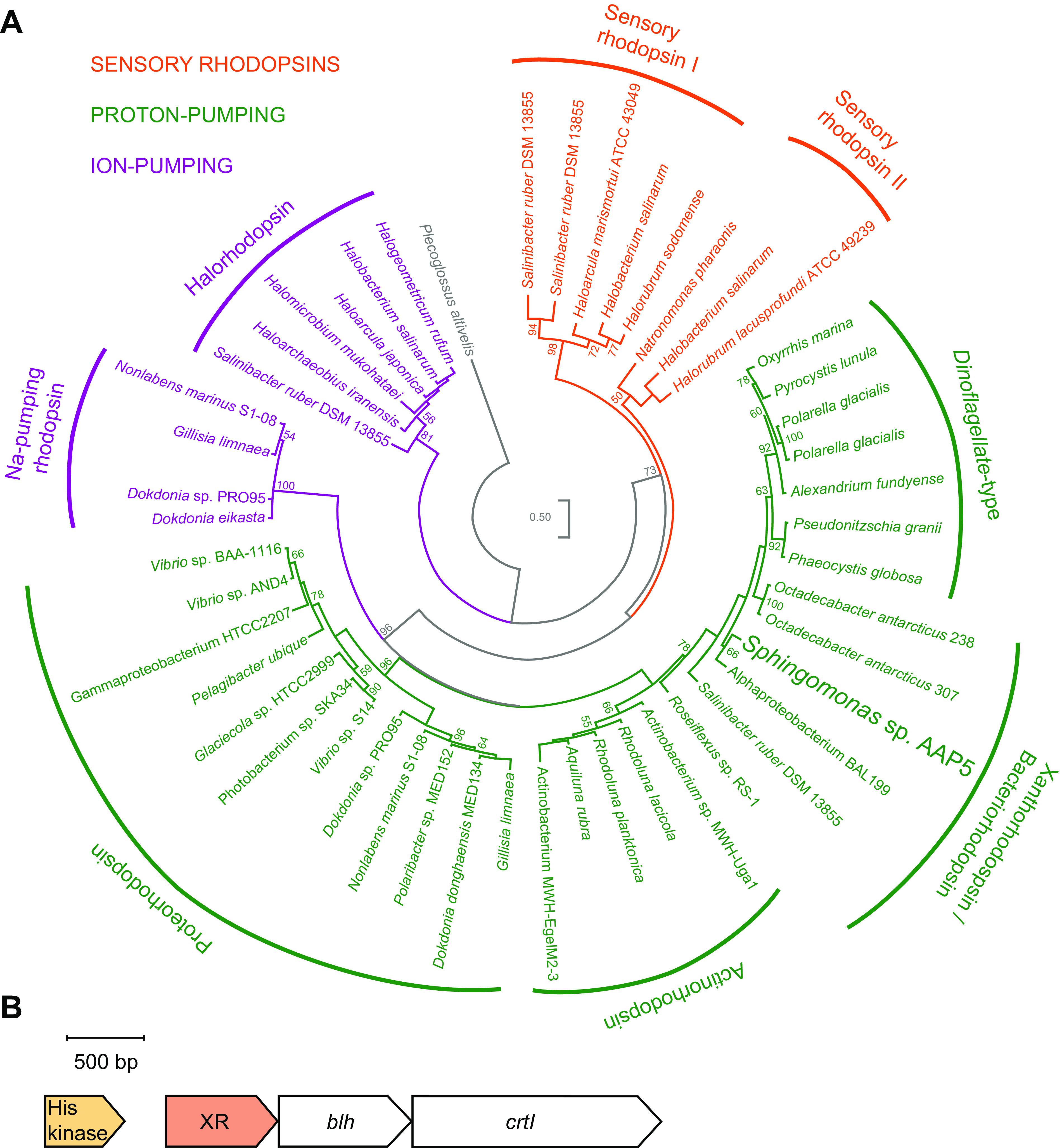
Phylogenetic affiliation and genomic context of the XR gene from the AAP5 strain. (A) Phylogenetic tree based on the alignment of amino acid sequences of the rhodopsin proteins from the three domains of life. XR sequence from the studied strain, which is highlighted by a bigger font, clearly clusters with other proton-pumping rhodopsins. The phylogenetic tree was calculated using the maximum likelihood (ML) algorithm with the LG model and bootstrap 100×. Plecoglossus altivelis was used as an outgroup. The bar represents the number of changes per position. Bootstrap values of >50% are shown. (B) Genomic environment of the XR gene. The bar represents sequence length in base pairs.

In addition to genes coding BChl *a*-containing reaction centers, we identified a single gene (E2E30_05030) coding for rhodopsin with 255 amino acid residues and seven transmembrane α-helices (Fig. S2A). Conserved amino acid positions of aspartate (Asp_92_) and glutamic acid (Glu_103_) on the 3rd helix (Fig. S2A) represented a presumable proton acceptor and donor, respectively, from a Schiff base in the proton transfer reactions during the rhodopsin photocycle (46). By the same token, the presence of leucine at the positions 99 and 100 on the 3rd helix (Fig. S2A) suggests that the identified rhodopsin absorbs green light (2, 47). Furthermore, the position of conserved glycine (Gly_150_ [Fig. S2B]) spoke for a tentative ability to form the keto-carotenoid binding site (48). Phylogenetic analysis supported that the identified rhodopsin belongs to the XR group of proton-pumping rhodopsins (Fig. 3A).

10.1128/mSystems.01044-20.2 FIG S2 (A) Prediction of transmembrane helices for the xanthorhodopsin protein from the AAP5 strain. The plot shows the posterior probabilities of an inside/outside/transmembrane helix for the amino acid sequence. For this protein, the single most probable topology clearly turns out to have seven transmembrane helices, the conserved feature of rhodopsins. Conserved amino acid positions of aspartate (Asp_92_) and glutamic acid (Glu_103_) on the 3rd helix, representing a presumative proton acceptor and donor, respectively, are highlighted in black. The two positions of conserved leucine (Leu_99_ and Leu_100_), which are predicted to be characteristic for rhodopsins with green light absorption maximum, are highlighted in green. The analysis was done using the TMHMM prediction tool (http://www.cbs.dtu.dk/services/TMHMM/). (B) Alignment of the XR sequences from the AAP5 strain and Salinibacter ruber. The position of conserved glycine (Gly_156_ in XR of *S. ruber*), which tentatively interacts with the keto-carotenoid, is highlighted in red. Download FIG S2, EPS file, 1.5 MB. Copyright © 2020 Kopejtka et al. 2020 Kopejtka et al. This content is distributed under the terms of the Creative Commons Attribution 4.0 International license.

Genomic environment analysis of the rhodopsin gene revealed that XR is in one operon with the β-carotene 15,15′-monooxygenase, encoded by the *blh* gene (E2E30_05025) (Fig. 3B). This enzyme converts β-carotene, which is a precursor for the biosynthesis of carotenoids, to two molecules of retinal. Retinal serves as a chromophore for the XR. The other *crt* genes (E2E30_15535, E2E30_15540, and E2E30_15550) necessary for the β-carotene synthesis from geranylgeranyl pyrophosphate (49) were found together in one gene cluster in other parts of the AAP5 genome.

Furthermore, the XR gene was in one gene cluster with a blue-light-activated histidine kinase (E2E30_05035) (Fig. 3B). To explore this further, we performed a phylogenetic analysis of XR sequences and their surrounding genes from other *Sphingomonas* representatives (Fig. S3). We found that the genomic neighborhood of the XR gene was completely syntenic in some *Sphingomonas* species (Fig. S3), forming a distinct phylogenetic group. Interestingly, this group contains representatives with and without PGC.

10.1128/mSystems.01044-20.3 FIG S3 Comparative phylogenetics of the XR from the AAP5 strain. Species (highlighted in blue) with the conserved genomic environment of the XR gene identical to that of the AAP5 strain (marked with an asterisk) form a distinct phylogenetic group, marked by the blue vertical bar. The blue scale bar represents sequence length in base pairs. The phylogenetic tree was based on the alignment of amino acid sequences of the XR protein from *Sphingomonas* representatives and calculated using both neighbor-joining (NJ) and maximum likelihood (ML) algorithms and 250× bootstrap replicates. Pseudorhodobacter antarcticus was used as an outgroup organism. The black scale bar represents number of changes per position. NJ/ML bootstrap values >50% are shown. Species with PGC are shown in bold type. Download FIG S3, EPS file, 1.6 MB. Copyright © 2020 Kopejtka et al. 2020 Kopejtka et al. This content is distributed under the terms of the Creative Commons Attribution 4.0 International license.

### Functional characterization of xanthorhodopsin.

**FIG 4:**
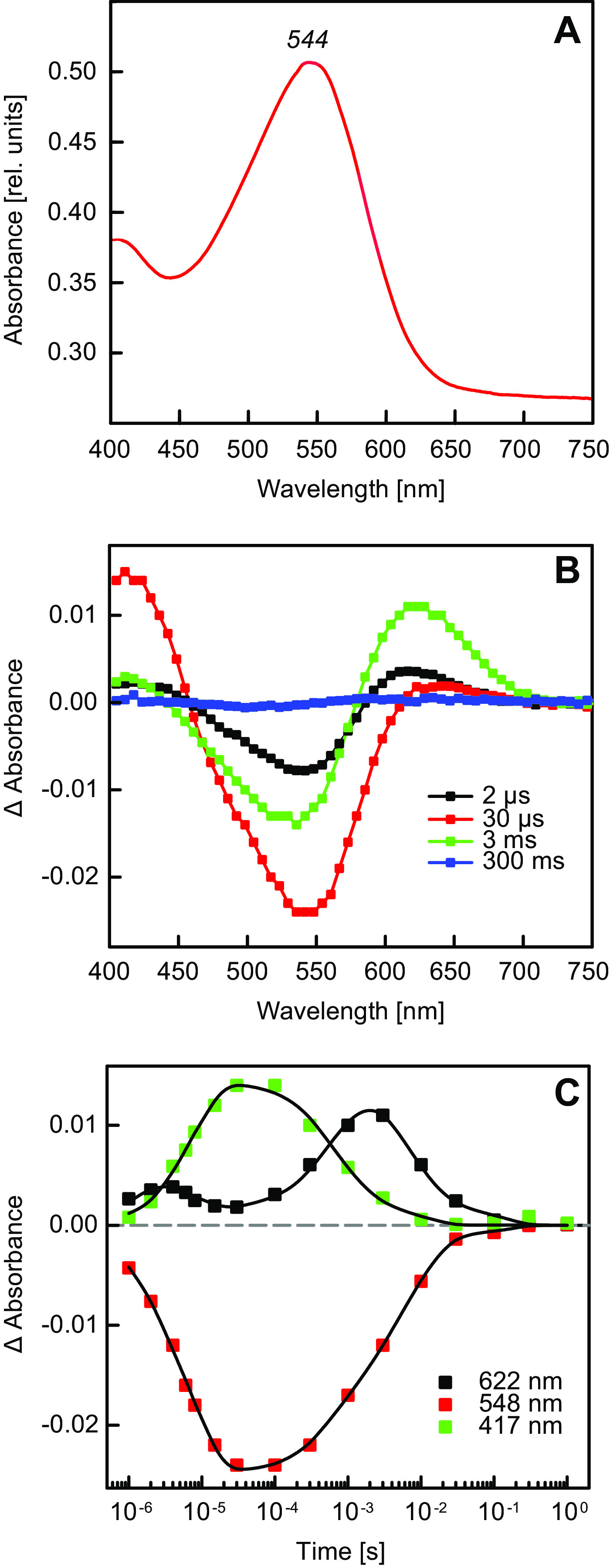
Spectroscopic analyses of xanthorhodopsin (XR). (A) Absorption spectrum of the E. coli-expressed XR gene. The absorption maximum of this recombinant protein was located in the green part of the spectrum at 544 nm. (B) Flash-induced changes in absorption spectra recorded at various delays after the actinic pulse. Delays are given in the legend. (C) Kinetics of absorbance changes at selected wavelengths following the actinic pulse (points). Traces given in solid lines show the results of the global fit of the kinetic data.

Despite the fact that XR gene was present in AAP5 genome, no rhodopsin proteins were identified in the collected cell membranes. Also, no traces of salinixanthin (carotenoid associated with xanthorhodopsins) was found in pigment extracts (Fig. S1). Therefore, we heterologously expressed the protein in Escherichia coli cells. After induction and retinal amendment, the recombinant strain displayed a weak pink color. The purified strep-tagged protein revealed an absorption maximum at 544 nm (Fig. 4A). The heterologously expressed XR was further analyzed by time-resolved absorption spectroscopy. As shown in Fig. 4B, the pulsed excitation led to a pronounced transient negative band with a maximum at ∼550 nm, this feature was flanked with positive absorption features peaking at ∼420 and 620 nm. The signal lacked pronounced vibrational structure as expected for retinal. Kinetics of the absorption changes at selected wavelengths can be found in Fig. 4C. This figure illustrates that the temporal development of the absorption features was complex: the global analysis of the time-resolved data required four components for satisfactory description, yielding time constants (±standard deviation) of 7.3 (±0.1) μs, 725 (±16) μs, 7.5 (±0.2) ms, and 93 (±8) ms. The absorption changes decayed completely on the ∼100-ms time scale. Our data on XR from the AAP5 strain compare well to the data obtained on XR from Salinibacter ruber (13) and demonstrate that the XR gene codes for a photoactive rhodopsin protein.

### Transcription of photosynthetic genes in AAP5 during growth on solid media and in liquid media.

**FIG 5:**
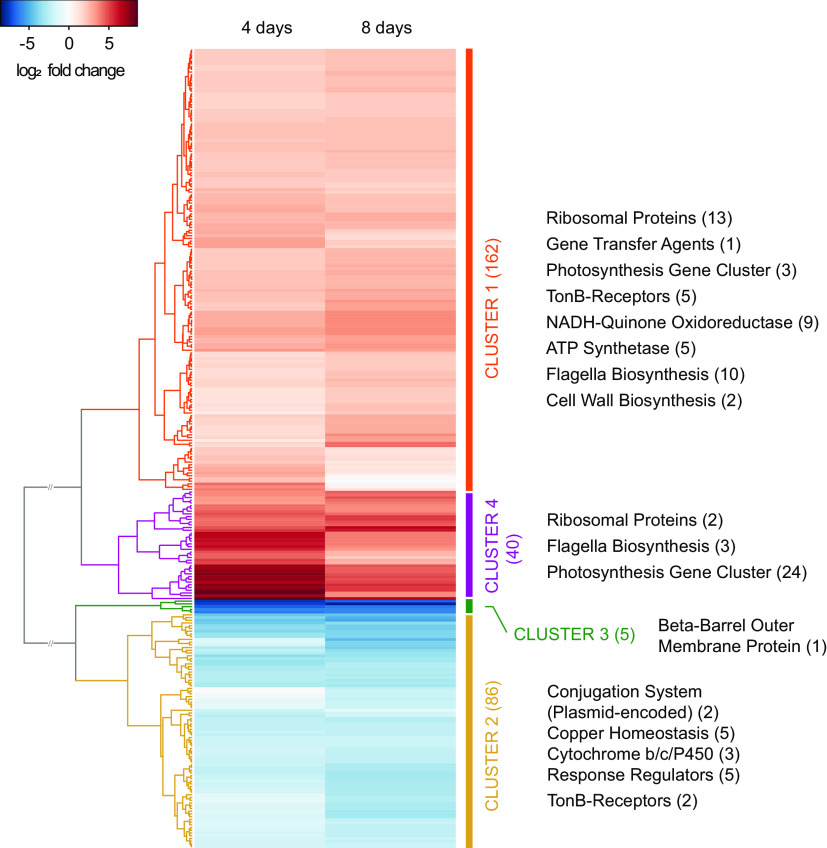
Transcriptome dynamics during growth on solid medium compared to growth in liquid medium. Heatmap visualization of log_2_ fold change for two time points grown on agar plate (4 and 8 days) compared to growth in liquid medium (control treatment). A total of 293 regulated genes are clustered into four main groups (clusters 1 to 4). For each cluster, the main groups of up- or downregulated genes are shown. Numbers in parentheses show the number of genes represented in each gene cluster/group. Cutoff values used for the analysis: *P* value = 0.05; log_2_ fold change > 2. FC.

The AAP5 strain had been cultured on agar plates where it showed a clear infrared emission of BChl *a*. However, when it was transferred to a liquid medium rich in organic nutrients, containing 42 mM organic carbon (OC), the cells contained no detectable BChl *a*. To understand this change in the BChl *a* content, we compared the transcriptome of cells grown for 4 days (when BChl *a* was first detectable) and 8 days (when BChl *a* was clearly visible) on agar plates with cells from liquid cultures after reaching their maximum optical density. All samples were harvested 4 h after the switch to dark phase of cultivation. Using a *P* value of <0.05 and log_2_ fold change (FC) of >2, we identified 293 up- and downregulated genes in the plate-grown samples that clustered into four main groups (Fig. 5).

The most upregulated genes were those of the PGC, and a gene coding for one out of four TonB energy transducer proteins (E2E30_15125). Five genes coding for TonB-dependent receptors were also upregulated, although to a lesser extent. Genes coding for the transcription-translation machinery and flagella were also upregulated. When comparing cells harvested after 4 days with those harvested after 8 days, the most upregulated genes from the earlier time point generally had higher transcription levels than those from the later time point.

The genes most downregulated during growth on solid medium were located in two adjacent putative operons (E2E30_RS03120-E2E30_RS03160) coding, among others for two response regulators and a transcription factor. A complete list of up- and downregulated genes can be found in Table S4. The rhodopsin and neighboring genes had only a few reads mapped under these conditions and thus were considered almost silent.

### Expression of the *pufM* gene under nutrient-limiting conditions.

The upregulation of PGC genes in the cells grown on the solid medium could have had three possible explanations. (i) They are expressed only when the cells grow as biofilm, (ii) Their expression is activated under low-oxygen environment formed inside the colonies. (iii) The expression of PGC genes is triggered by nutrient limitation. The last explanation was additionally supported by the activation of TonB transport components along with the PGC. To test these options, we performed an experiment with AAP5 cells grown in well-aerated liquid cultures with lowered concentrations of organic carbon sources (glucose, pyruvate, yeast extract, and peptone).

Since BChl *a* synthesis was not observed in cultures grown in full medium (i.e., 42 mM OC), we considered the relative expression of *pufM* at this concentration of organic components as repressed. This was confirmed using reverse transcription quantitative PCR (RT-qPCR) (Fig. S4, top panel). *pufM* gene expression was first observed in cultures grown in medium with >5-fold dilution of organic nutrients and reached its maximum at 15-fold dilution. To further investigate this phenomenon, batch cultures were grown at a 10-fold-reduced concentration of organic nutrients until the stationary phase, and then we amended the cultures with either organic carbon (glucose), nitrogen (NH_4_Cl), or iron (FeCl_3_) at concentrations corresponding to one-third of those in the full medium. For normalization, we used control samples (without any amendment). After 24 h of the amendments, the *pufM* relative expression decreased 33-fold in cultures amended with glucose (Tukey test, *P* value = 0.0222, *t* value = −3.781), 2-fold in cultures supplemented with nitrogen (*P* value = 0.8497, *t* value = −0.807), and 1.8-fold in iron-amended treatments (*P* value = 0.9120, *t* value = −0.652). After 48 h of glucose amendment, the *pufM* relative expression recovered to the level corresponding to 85% of *pufM* relative expression in the control treatment (*P* value = 0.996, *t* value = −0.226). For nitrogen and iron, these values were lower by 66% (*P* value = 0.932, *t* value = −0.589) and 21% (*P* value = 0.264, *t* value = −2.027), respectively (Fig. S4, bottom panel). These results support our hypothesis that *pufM* expression is repressed by high concentrations of glucose.

10.1128/mSystems.01044-20.4 FIG S4 Transcriptional dynamic of the *pufM* gene under nutrient-limiting conditions. (Top) Influence of organic carbon (OC) concentration on the transcription of the *pufM* gene. In cultures grown in organic medium with OC dilution of >5×, *pufM* gene transcription was significantly upregulated. The OC concentration of 100% corresponds to the OC concentration (42 mM) in full organic medium. (Bottom) Cultivation of the AAP5 strain in organic medium with low-glucose concentration (corresponding to glucose dilution 10×) triggers the *pufM* gene expression (time point 0), whereas the increase of glucose concentration in the organic medium (corresponding to 1/3 of OC concentration in full organic medium) effectively inhibits it (time point 1). Forty-eight hours after the glucose amendment, *pufM* relative expression recovered to a level similar to that in the control treatment, whereas for nitrogen and iron amendment, the changes in *pufM* relative expression were not statistically significant (time points 1 and 2). The Δ*Ct* mean values and standard deviations calculated from three parallel biological replicates are shown. *Ct*, threshold cycle. Download FIG S4, EPS file, 1.4 MB. Copyright © 2020 Kopejtka et al. 2020 Kopejtka et al. This content is distributed under the terms of the Creative Commons Attribution 4.0 International license.

### Transcriptome response to low-carbon conditions.

**FIG 6:**
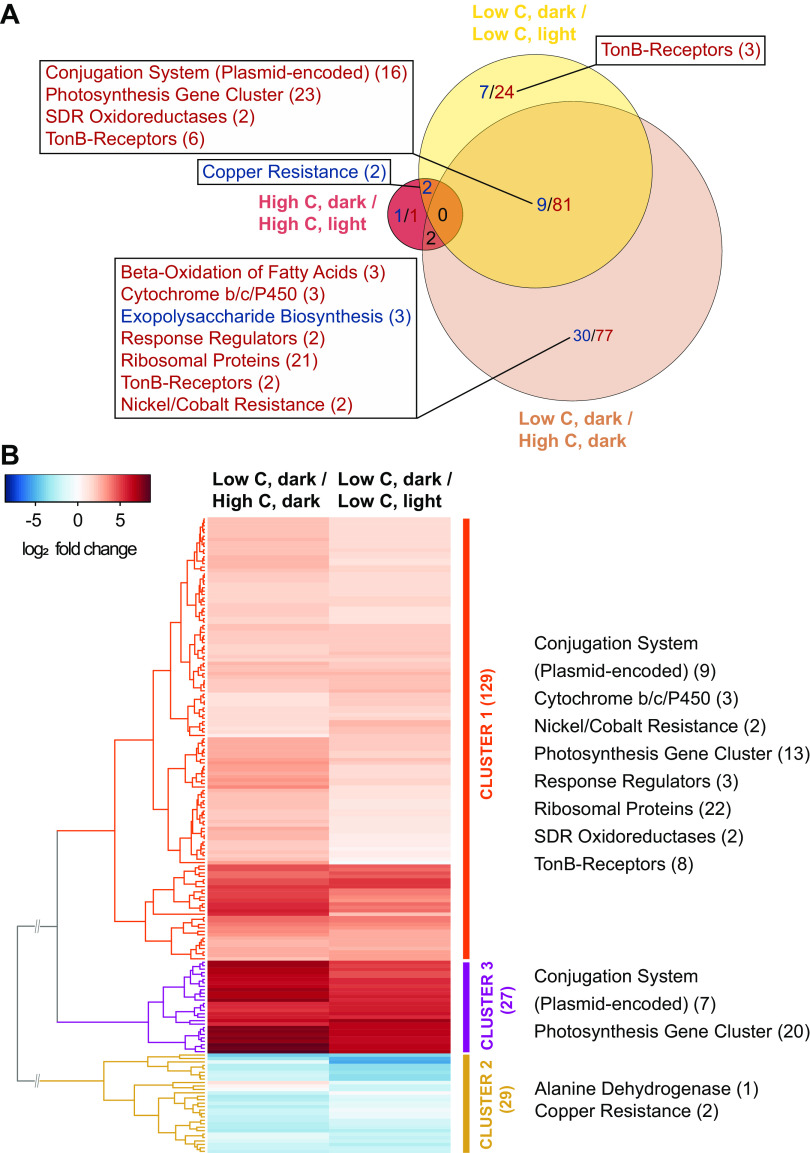
Transcriptome dynamics during growth in liquid medium under carbon-limiting conditions and different light regimes. (A) Overlap of genes that were significantly regulated for each combination of experimental and control treatments. Groups of genes with common function are highlighted. Upregulated genes are in red, downregulated genes are in blue. Numbers in parentheses show the number of genes represented in each gene group. Cutoff values used for the analysis: *P* value = 0.05; no log_2_ fold change (FC) cutoff value. (B) Heatmap visualization of log_2_ FCs between two experimental treatments both grown and harvested under the same conditions (cells grown in carbon-deficient medium, harvested in dark) but compared to two different control treatments (cells grown in carbon-rich medium, harvested in dark and cells grown in carbon-deficient medium, harvested in light). In total, 185 regulated genes are clustered into three main groups (clusters 1 to 3). For each cluster, the main groups of up- or downregulated genes are shown. Numbers in parentheses show the number of genes represented in each gene cluster/group. Cutoff values used for the analysis: *P* value = 0.05; log_2_ FC > 2.

To characterize differences in gene expression between cells grown under carbon-limited and carbon-replete conditions on the whole-transcriptome level, we cultivated the AAP5 strain in 10-fold-diluted and full liquid medium. To analyze the influence of light on PS gene expression, we sampled during both phases of the 12-h dark/12-h light cultivation (4 h after the respective switch). With high-carbon concentration, light had only a minimal influence on gene expression. In contrast, with low-carbon concentration, light had a significant effect on gene expression (Fig. 6A). Genes that responded to light under low-carbon conditions showed a considerable overlap with those that were differentially expressed under low-carbon compared to high-carbon conditions (Fig. 6A).

Due to the negligible influence of light at high-carbon conditions, this sample was not further considered. We compared log_2_ FCs between samples from the carbon-deficient medium harvested in the dark with samples from the carbon-rich medium, harvested in the dark and samples from carbon-deficient medium, harvested in the light. Using a *P* value of <0.05 and log_2_ FC of >2 resulted in a total number of 185 genes (158 up- and 27 downregulated) that clustered into three main groups (Fig. 6B).

Among the most upregulated genes were the complete PGC and the full plasmid-encoded type IV secretion system gene cluster, which probably mediates the conjugational transfer of its host replicon. Eight TonB-dependent receptor genes and genes coding for the transcription-translation machinery were also upregulated.

The most downregulated gene under both conditions coded for the alanine dehydrogenase (E2E30_RS03695; EC 1.4.1.1). In bacteria, this enzyme is crucial for the utilization of l-alanine as an energy source and is also involved in the transcriptional regulation of alanine metabolism (50). A complete list of up- and downregulated genes can be found in Table S5. Again, the XR gene was silent under all conditions tested.

However, there are four organic components (glucose, sodium pyruvate, peptone, and yeast extract) potentially contributing to the repression of *pufM* expression in the organic medium we used. To find out whether this repression is caused specifically by glucose, we grew batch cultures with a reduced concentration of organic nutrients (dilution 10×) until the stationary phase, and then we amended the cultures with OC in the form of either glucose, sodium pyruvate, peptone, or yeast extract at concentrations corresponding to one-third of those in the full medium. For normalization, we used control samples (without any amendment). After 24 h of the amendments, *pufM* relative expression decreased 296-fold in cultures amended with glucose (Tukey test, *P* value =  0.0003, *t* value = −6.926), 3.8-fold in cultures supplemented with sodium pyruvate (*P* value = 0.518078, *t* value = −1.620), 1.6-fold in cultures supplemented with peptone (*P* value = 0.980792, *t* value = −0.540), and 1.2-fold in yeast-extract-amended treatments (*P* value = 0. 998834, *t* value = −0.259). After 48 h of the glucose amendment, *pufM* relative expression recovered to the level corresponding to 69% of *pufM* relative expression in the control treatment (*P* value = 0. 9548, *t* value = −0.684). However, in cultures supplemented with sodium pyruvate, peptone, or yeast extract, *pufM* relative expression increased 1.9-fold (*P* value = 0. 7550, *t* value = 1.193), 1.5-fold (*P* value = 0. 9454, *t* value = 0.723), and 3.9-fold (*P* value = 0. 0.1571, t value = 2.570), respectively (Fig. S5). These results show that out of four sources of OC (glucose, sodium pyruvate, peptone, and yeast extract), only glucose significantly repressed *pufM* expression.

10.1128/mSystems.01044-20.5 FIG S5 Influence of different sources of organic carbon (OC) on the *pufM* gene transcriptional dynamic. Among four sources of OC tested, only the addition of glucose (in an amount corresponding to 1/3 of OC concentration in full organic medium) effectively inhibited the *pufM* gene expression (time point 1). Forty-eight hours after the glucose amendment, the *pufM* relative expression recovered to a level similar to that in the control treatment. For amendment of other sources of OC, the changes in *pufM* relative expression were not statistically significant (time points 1 and 2). The Δ*Ct* mean values and standard deviations calculated from three parallel biological replicates are shown. *Ct*, threshold cycle. Download FIG S5, EPS file, 1.3 MB. Copyright © 2020 Kopejtka et al. 2020 Kopejtka et al. This content is distributed under the terms of the Creative Commons Attribution 4.0 International license.

Intrigued by this phenomenon, we wanted to confirm previous results not only at the expression level but also with regard to the actual formation of the photosynthetic machinery. To monitor BChl *a* biosynthesis, we cultivated the AAP5 strain in the full-strength organic medium where glucose was substituted by either galactose, rhamnose, or pyruvate in the amount corresponding to the carbon content of glucose in full medium. For a control, we used cells grown in the medium containing glucose. During 8 days of cultivation, we detected BChl *a* only in cultures with pyruvate and rhamnose with a maximum 1.39 × 10^−4^ and 2.22 × 10^−4^ g (Bchl *a*) g (protein)^−1^, respectively. This documents that Bchl *a* synthesis is inhibited also by structural analogues of glucose, such as galactose.

## DISCUSSION

### Control of AAP expression.

While PGC or rhodopsin genes have been found in many bacterial species, the simultaneous presence of both systems for harvesting light energy in one organism is unique. Our results document that under low-glucose conditions, the cells assemble fully functional photosynthetic core complexes containing BChl *a* and spirilloxanthin as an auxiliary pigment. The absence of the peripheral light-harvesting complex LH2 is relatively common among AAP bacteria (8).

In contrast to the closely related phototrophic purple nonsulfur bacteria performing anaerobic anoxygenic photosynthesis, AAP bacteria do not respond to oxygen by shutting down their PS apparatus. On the contrary, oxygen is strictly required for BChl *a* synthesis in these bacteria (8). Our transcriptomic data suggest that expression of PS genes in AAP bacteria may be inhibited not only by light, as documented earlier (42, 51), but also by higher concentrations of OC. A similar result was reported for the freshwater AAP bacterium Roseateles depolymerans (52). However, this study was focused only on the expression of the *puf* operon. These authors concluded that transcription of the *puf* genes is controlled by changes not only in light intensity and oxygen tension but also in carbon sources (52).

While Suyama and coworkers (52) argued that Roseateles depolymerans upregulates its *puf* operon under low-OC conditions, our study shows that in photoheterotrophic AAP5, the photosynthetic apparatus is specifically repressed by glucose and galactose but not rhamnose, pyruvate, or complex C sources. The presence of monosaccharides might be indicative of the presence of OC in general and signals to the bacterium that costly biosynthesis of the photo-apparatus is not needed. Under low-OC availability, photoheterotrophic bacteria generate energy from light to decrease its carbon demand (9). This trophic strategy may be beneficial especially for opportunistic species such as *Sphingomonas*. The marine AAP strain Dinoroseobacter shibae uses this energy to generate ATP (53) to increase its biomass yield (10). The simultaneous transcriptional activation of the PGC and TonB transport system in AAP5 suggests a potential additional utilization of light energy. TonB-dependent transporters (TBDTs) exploit the proton motive force for the import of nutrients across the outer membrane into the periplasmatic space. TBDTs were initially discovered as transporters for iron-siderophore complexes. However, it is now clear that some representatives of TBDTs can import a variety of nutrients, including vitamins but also carbohydrates (reviewed by Noinaj and coworkers [54]). Recently, lignin-derived aromatic compounds have been identified as novel substrates for a TBDT of *Sphingobium* sp. strain SYK-6 (55). *Sphingomonadaceae* from extreme oligotrophic environments often encode large numbers (up to 134) TBDTs in their genomes (56). The genome of AAP5 contains 64 TonB-dependent receptor proteins. To thrive in an oligotrophic environment such as Gossenköllesee, AAP5 may use the proton gradient to concentrate scarce nutrients in the periplasm from where they can be imported into the cell. Light-driven import of thiamine via a TBDT has indeed been suggested for proteorhodopsin-containing flavobacterium *Dokdonia* sp. MED134 and DSW1 (18).

### Rhodopsin functionality.

The XR gene seemed to be fully functional, and the complete pathway for the synthesis of its chromophore retinal is present in the genome. However, it did not contain a *crtO* gene, coding for the carotenoid antenna of the XR. Transmembrane domain analysis of the XR gene predicts that the putative protein contains seven transmembrane domains, which is a conserved hallmark of all rhodopsins. Its amino acid sequence clustered with other known XR genes with strong statistical support (78% maximum likelihood [ML] bootstrap). The presence of characteristic conserved amino acid residues (2, 47, 48) in the sequence suggested that the identified rhodopsin absorbs green light and can tentatively interact with the keto-carotenoid. The former was confirmed when transformed E. coli cells overexpressing this rhodopsin gene exhibited an absorption maximum in the green region of the absorption spectrum, and the latter when E. coli displayed a characteristic pink color after the retinal amendment. Both changes demonstrated that the heterologously expressed rhodopsin protein was properly folded and functional.

In contrast to the recombinant E. coli strain overexpressing the rhodopsin gene, we were not able to detect any absorption peak of rhodopsin in the membrane fraction during the purification of PS complexes. This is in agreement with the fact that the identified rhodopsin gene was virtually not expressed under experimental conditions.

The flash-induced transient absorption data confirm that the product of the XR gene is capable of performing the photocycle. Our analysis of the kinetic data resolved four components with time constants in the microsecond-to-millisecond range. The fastest process resolved had a time constant of 7.3 μs, which is in perfect agreement with the data from XR of S. ruber (13). The slowest phase of the XR photocycle was found to be ∼100 ms, also in agreement with the cited work. In our case, the data do not support the resolution with a total of six kinetic components, as suggested by Balashov and coworkers (13). However, it should be noted that unlike our XR sample, the system studied in the cited work also contained the salinixanthin antenna pigment whose electrochromic response contributes to the complexity of the absorption data.

### Why are there two systems for light harvesting?

The main question of why an organism keeps in its genome two different systems for capturing light energy remains. One possibility is that these systems work together, and the metabolic benefit for the organisms is higher than when using only one of the systems. One such cooperation is found during oxygenic photosynthesis, where the coupled action of two photosystems makes it possible to bridge large redox potential necessary for extracting electrons from water (1). However, there is no support for this hypothesis in the case of *Sphingomonas* sp. AAP5, since no xanthorhodopsin was found in the membranes together with BChl *a*-containing photosystems. The other option is that AAP5 utilizes the two systems under different conditions. We showed that BChl *a*-containing photosystems are used under low-glucose conditions. There may be some very specific conditions, which are not suitable for bacteriochlorophyll and where the bacterium could use the rhodopsin system, but it is very difficult to test this hypothesis under laboratory conditions. Although we did not observe XR expression in our experiments, it is certainly possible that there is a specific physiological condition or external stimulus that induces its synthesis.

On the other hand, there is good reason to believe that XR in AAP5 may have another function than light harvesting. Phylogenetic inference placed the XR sequences on the same branch as nonbacterial dinoflagellate-type rhodopsins. In the marine dinoflagellate Prorocentrum donghaiense, this phylogenetic affiliation was recently confirmed, although it seems these two groups of rhodopsins have a distinct evolutionary origin. Sensory rhodopsins were found to accompany chlorophyll-based photosynthesis in cyanobacteria (22–25). It is hard to imagine that XR can provide a significant energy benefit for algae because they can rely on an effective energy source such as oxygenic photosynthesis. Moreover, AAP5 does not contain the *crtO* gene, coding for the carotenoid antenna of the XR, which significantly restricts its light absorption properties.

The XR gene of AAP5 is in one operon with a histidine kinase gene. Interestingly, the existence of histidine kinase rhodopsin (HKR) was recently reported for the marine alga Chlamydomonas reinhardtii (57). HKRs are modular proteins containing rhodopsin, a His kinase, a response regulator, and in some cases, an effector domain. All these lines of evidence indicate that the XR gene may have a function other than light energy harvesting. Therefore, we hypothesize that the rhodopsin might represent part of a light-sensing system, rather than a light-harvesting system covering cellular energy needs. The XR together with other numerous photoreceptors existing in this bacterium allows it to react to changes in incident light, an important environmental factor in the sunlit waters from which this strain was isolated.

## MATERIALS AND METHODS

### Strain isolation.

One microliter of the lake water sample was diluted into 100 μl of sterile half-strength R2A medium, and the dilution was spread onto half-strength standard R2A agar plates (DSMZ medium 830). Agar plates were incubated aerobically at 25°C under 12-h-light/12-h-dark cycles until colonies were visible, which were then screened for the presence of BChl *a* using an infrared (IR) imaging system (58). IR-positive colonies were repeatedly streaked onto new agar plates until pure cultures were obtained. The *Sphingomonas* sp. strain AAP5 has been deposited in the DSMZ under the number DSM 111157.

### Cultivation conditions.

Initially, the strain was grown on R2A solid medium (DSMZ medium 830). Later the medium was modified by adding 1 g NaCl liter^−1^, which significantly enhanced growth. For the detailed composition of the modified media used for the nutrient limitation experiments, see Table S1 in the supplemental material. Cultures were incubated aerobically in 100 ml of the appropriate medium in 250-ml flasks with cotton plugs on an orbital shaker (150 rpm) at 22°C. Illumination was provided by a bank of Dulux L 55W/865 luminescent tubes (Osram, Germany, spectral temperature of 6500 K) delivering ca. 100 μmol photons m^−2^ s^−1^. If not stated otherwise, the cultures were grown under a 12-h-dark/12-h-light regime. At the beginning of each experiment, the late-exponential-phase inoculum (optical density at 650 nm [OD_650_] of approximately 0.8) grown in full organic medium (Table S1) at 22°C in darkness was diluted to an OD_650_ of 0.01 with appropriate organic medium and distributed into Erlenmeyer flasks. The growth of these new cultures was monitored several times per day by turbidity measurements at 650 nm. For *pufM* transcription experiments, sampling was done 4 h after the switch to the dark phase of cultivation.

10.1128/mSystems.01044-20.6 TABLE S1 Organic media used for AAP5 cultivation. Download Table S1, PDF file, 0.2 MB. Copyright © 2020 Kopejtka et al. 2020 Kopejtka et al. This content is distributed under the terms of the Creative Commons Attribution 4.0 International license.

10.1128/mSystems.01044-20.7 TABLE S2 General characteristics of the AAP5 genome after NCBI PGAP. Hypo., hypothetical; PGC, photosynthesis gene cluster. Download Table S2, PDF file, 0.2 MB. Copyright © 2020 Kopejtka et al. 2020 Kopejtka et al. This content is distributed under the terms of the Creative Commons Attribution 4.0 International license.

The culture purity during the experiments was monitored with catalyzed reporter deposition-fluorescence *in situ* hybridization (CARD-FISH) (59) or the modified fluorescence *in situ* hybridization technique FISH IR that maintains BChl *a* autofluorescence (60), using the Sphingo-866 probe targeting *Sphingomonadales* (61).

### Genome sequencing.

Genomic DNA was extracted from 45 ml of culture by centrifugation at 13,000 × *g* and purified using the TIANamp genomic DNA kit (Tiangen Biotech [Beijing] Co., Ltd., China). DNA quantity and quality were determined using a NanoDrop 2000.

### (i) Illumina library preparation and sequencing.

The Illumina whole-genome shotgun sequencing was done using the Illumina HiSeq 2000 platform at Macrogen (Seoul, South Korea). Procedures for DNA shearing, library preparation and quality control, sample loading, and sequencer operation were performed according to Macrogen’s standard protocols.

### (ii) PacBio library preparation and sequencing.

Single-molecule real-time (SMRT) bell template library was prepared according to the instructions from the manufacturer (Pacific Biosciences, Menlo Park, CA, USA), following the Procedure & Checklist – Greater Than 10 kb Template Preparation. SMRT sequencing was carried out on a PacBio *RSII* (Pacific Biosciences, Menlo Park, CA, USA) taking one 240-min movie for one SMRT cell using P6 chemistry. Sequencing resulted in 87,520 postfiltered reads with a mean read length of 13,434 bp.

### (iii) Complete genome assembly and annotation.

SMRT Cell data were assembled using the “RS_HGAP_Assembly.3” protocol included in SMRT Portal version 2.3.0 using default parameters. The chromosome was circularized, particularly artificial redundancies at the ends of the contigs were removed and adjusted to *dnaA* as the first gene. Error correction was performed by mapping 7 million paired-end Illumina reads of 2 × 100 bp onto finished genomes using BWA (62) with subsequent variant and consensus calling using VarScan (63). A consensus concordance of QV60 was confirmed for the genome.

Annotation was done using the NCBI Prokaryotic Genome Annotation Pipeline (PGAP, released 2013, https://www.ncbi.nlm.nih.gov/genome/annotation_prok/). The genome sequence was deposited at NCBI under the accession numbers CP037913 (chromosome) and CP037914 to CP037916 (plasmids).

### Phylogenetic analyses.

16S rRNA gene sequences and amino acid sequences were obtained either from the SILVA database (64) or NCBI GenBank (March 2019) and aligned using ClustalW (65). Ambiguously aligned regions and gaps were manually excluded from further analysis. The 16S rRNA phylogenetic tree was computed using both neighbor-joining (NJ) (66) and maximum likelihood (ML) (67) algorithms included in the MEGA 6.06 software (68). The Tamura-Nei model (69) was used for inferring the NJ tree. The ML tree was constructed using the general tree reversible (GTR) nucleotide substitution model (70). A uniform rate of nucleotide substitution was used. Phylogenetic trees based on alignments of amino acid sequences were inferred using an ML algorithm with the LG model.

### Heterologous expression of XR in E. coli.

The XR gene (E2E30_05030) was cloned into pTD-C_eYFPTwinStrep plasmid (Addgene identifier [ID] 45942) using EcoRI and XbaI restriction sites. The resulting plasmid expressing XR as a twin-Strep-tagged protein was transformed into E. coli BL21 for expression. The fidelity of the construct was verified by sequencing. Expression was induced by 1 mM isopropyl-β-d-thiogalactopyranoside (IPTG) followed by the addition of 10 μM all-*trans* retinal (Sigma-Aldrich, Germany) and carried out for 6 h at room temperature. XR was localized in the membrane fraction. Induced cells were harvested by centrifugation (6,000 × *g* for 10 min at 4°C). The cell pellet was resuspended in 20 mM Tris-HCl buffer (pH 8.0) containing 0.5 mM EDTA, 3 mM MgCl_2_, and 50 U/ml Benzonase nuclease. To isolate membrane fraction containing heterologously expressed rhodopsin, cells were homogenized using EmulsiFlex-C5 (Avestin, Canada). Crude membranes were collected by ultracentrifugation (45,000 × *g* for 60 min at 4°C) and solubilized in 20 mM Tris-HCl buffer (pH 8.0) containing 2% dodecyl maltoside (DDM) and 0.5% Triton X-100. After centrifugation (13,000 × *g* for 20 min at 4°C), the supernatant was applied to a *Strep*-Tactin gravity flow column (IBA GmbH, Germany), and the recombinant rhodopsin was eluted by the addition of 2.5 mM desthiobiotin. The absorption spectrum of the purified rhodopsin was recorded using a Shimadzu UV 2600 spectrophotometer.

### Optical spectroscopy.

Steady-state absorption spectra were recorded using a double-beam spectrophotometer UV2600 (Shimadzu, Japan) equipped with an integrating sphere. Flash-induced transient absorption measurements were performed on a sample of heterologously expressed rhodopsin using a locally built flash photolysis instrument as described previously (71). The light-induced absorbance changes were driven by microsecond pulses monitored in the 400- to 700-nm range with microsecond temporal resolution over microsecond-to-second-long time delays. The transient absorption data were globally fit by a sum of exponential functions using Matlab (The MathWorks Inc. USA) scripts, and the confidence intervals of the resulting rate constants were estimated using bootstrap resampling as described previously (72).

### RNA sequencing. (i) RNA isolation and purification.

Cells were harvested by centrifugation. To each cell pellet, 1 ml of PGTX extraction solution (73) was added and pellets were immediately frozen in liquid nitrogen and stored at –70°C until extraction. RNA was extracted following the protocol by Pinto and colleagues (73). Briefly, samples were incubated at 95°C for 5 min and immediately placed on ice for 10 min. After the addition of 800 μl chloroform, the extraction mix was centrifuged to promote phase separation. The aqueous phase was then retrieved and mixed with an equal volume of chloroform, centrifuged, and retrieved again. RNA was precipitated with isopropanol overnight at –20°C, recovered by centrifugation, washed with 70% ethanol, air dried, and finally dissolved in an appropriate volume of sterile nuclease-free water. The RNeasy kit (Qiagen, the Netherlands) was used for purification according to the manufacturer’s manual. The first digestion of genomic DNA was performed on the column, using DNase I (Qiagen, the Netherlands) according to the manufacturer’s protocol. Total RNA was eluted in 88 μl RNase-free H_2_O, and the second DNase I digestion was made in solution, followed by a second RNeasy purification step, which included an additional washing step with 80% ethanol done before elution with 30 μl RNase-free water. Samples were tested for genomic DNA contamination by using RNA directly as a template for PCR. Possible contaminating DNA was removed using the TURBO DNA-free kit (Ambion) according to the manufacturer’s protocol.

### (ii) Illumina library preparation and sequencing.

rRNA (16S and 23S) was removed from total RNA using the RiboZero kit (Illumina, San Diego, CA, USA) according to the manufacturer’s protocol. Single-end, strand-specific cDNA libraries were prepared using the Scriptseq v2 RNA-Seq library preparation kit (Illumina, San Diego, CA, USA) according to the manufacturer’s protocol. For sequencing, equal volumes of libraries (12 pM) were multiplexed on a single lane. Cluster generation was performed with cBot (Illumina, San Diego, CA, USA) using TruSeq SR Cluster kit v3–cBot-HS (Illumina, San Diego, CA, USA). Sequencing was done on the HiSeq 2500 (Illumina, San Diego, CA, USA) using TruSeq SBS kit v3 - HS (Illumina, San Diego, CA, USA) for 50 cycles. Image analysis and base calling were performed using the Illumina pipeline v 1.8 (Illumina, San Diego, CA, USA).

The sequencing output (50-bp single end) was processed using the FASTQ-mcf suite (https://github.com/ExpressionAnalysis/ea-utils). Low-quality bases (Phred score < 30) and Illumina adapters were clipped. Reads were mapped to the genome of strain AAP5 using bowtie2 (74) with default parameters for single-end reads. The resulting sam files were converted to an indexed binary format using samtools (62); featureCounts (75) was used to count the reads mapping to genes. Quality control of biological replicates and statistical analysis were performed in the R environment using the package edgeR (76).

### Design and optimization of specific primers.

To investigate photosynthesis gene expression, we used the *pufM* gene, which encodes the M subunit of the bacterial reaction centers (77, 78). The RNA polymerase δ subunit gene (*rpoD*) was used as reference gene for the RT-qPCR. Both specific primer pairs were designed to produce 150- to 200-bp-long partial sequences. The primers were designed with 50 to 60% GC content to ensure similar melting temperature characteristics. The primers were first tested with genomic DNA as the template, and the identity of PCR products was confirmed using Sanger sequencing. Primers and PCR conditions are listed in Table S3.

10.1128/mSystems.01044-20.8 TABLE S3 Primers and RT-PCR conditions. Download Table S3, PDF file, 0.2 MB. Copyright © 2020 Kopejtka et al. 2020 Kopejtka et al. This content is distributed under the terms of the Creative Commons Attribution 4.0 International license.

10.1128/mSystems.01044-20.9 TABLE S4 A complete list of up- and down-regulated genes when AAP5 cells were grown on solid media and in liquid media. Download Table S4, XLSX file, 0.5 MB. Copyright © 2020 Kopejtka et al. 2020 Kopejtka et al. This content is distributed under the terms of the Creative Commons Attribution 4.0 International license.

10.1128/mSystems.01044-20.10 TABLE S5 A complete list of up- and down-regulated genes when AAP5 cells were grown under low-carbon conditions. Download Table S5, XLSX file, 0.7 MB. Copyright © 2020 Kopejtka et al. 2020 Kopejtka et al. This content is distributed under the terms of the Creative Commons Attribution 4.0 International license.

### RT-qPCR.

Total RNA (130 ng) was reverse transcribed using the Transcriptor First Strand cDNA Synthesis kit (Roche) according to the manufacturer’s protocol in a total volume of 20 μl with 30 min at 55°C for cDNA synthesis. Relative quantification was performed in triplicate in CFX Connect RT PCR Detection System (BioRad, USA) in 20-μl reaction mixtures containing 1× PowerUp Sybr green master mix (Applied Biosystems, USA), 8 pmol of each primer, and 2 μl of 4× diluted cDNA. The *rpoD* gene was used as the reference gene. For all primers used, the amplification efficiency was determined by qPCR with a serial dilution of pooled samples. The comparative threshold cycle (*C_T_*) method (79) was used to quantify the fold changes in gene espression. Differences in *pufM* expression between different amendments and control treatment were tested using two-way analysis of variance (ANOVA) and *post hoc* Tukey test in the R environment (version 3.6.2) and multcomp package (version 1.4.13). Primers and PCR conditions are listed in Table S3.

### Pigment analysis.

Pigments were analyzed using a high-performance liquid chromatography system Nexera LC-40 HPLC system (Shimadzu Inc., Japan) equipped with a diode array UV-visible (UV-VIS) detector. The cells were collected by centrifugation (10,000 × *g* for 2 min), and the pellet was gently resuspended in 20 μl of water and then extracted with 1 ml of 100% methanol. The pigments were separated with heated (40°C) Phenomenex Luna 3μC8(2) 100-Å column, using a binary solvent gradient (solvent A) 28 mM ammonium acetate in water:methanol (1:3 [vol:vol]) and (solvent B) 100% methanol at a flow rate of 0.8 ml min^−1^. The eluted pigments were identified based on their absorption spectra and retention times.

### Analysis of PS complexes.

AAP5 cells were grown aerobically at 22°C in 2 liters of organic medium with 10× dilution of organic components (Table S1). After reaching maximum OD_650_ (∼0.1), the culture was harvested by centrifugation (6,000 × *g* for 10 min), yielding ca. 1 g of wet biomass. The cells were broken using an EmulsiFlex-C5 (Avestin Inc., Canada) at 140 MPa, and unbroken cells with cell debris were removed by centrifugation for 10 min at 5,000 × *g*. The released membranes were collected by ultracentrifugation (60 min, 110,000 × *g*). The PS complexes were purified by ion-exchange and size exclusion chromatography as described before (45).

For the electron microscopy analysis, the freshly prepared PS complexes were deposited on glow-discharged carbon-coated copper grids, negatively stained with 1.5% uranyl acetate, and visualized using a JEOL JEM–2100F transmission electron microscope (JEOL, Tokyo, Japan; using 200 kV at 30,000 × magnification). Transmission electron microscopy (TEM) images were recorded using a bottom-mounted Gatan charge-coupled-device (CCD) Orius SC1000 camera, with a resolution corresponding to 2.23 Å per pixel. Image analysis was carried out using RELION (80). The selected projections were rotationally and translationally aligned and treated by an empirical Bayesian approach in combination with a classification procedure to refine two-dimensional (2D) class averages.

### Data availability.

The complete genome sequence is deposited at NCBI GenBank under the following accession numbers: CP037913 (chromosome) and CP037914 to CP037916 (plasmids). Raw and processed RNA sequencing data are available from the Gene Expression Omnibus database under accession numbers GSE147049 and GSE147051.
