# Supplementary material for: Simultaneous Presence of Bacteriochlorophyll and Xanthorhodopsin Genes in a Freshwater Bacterium
Source: mSystems. 2020 Dec 22;5(6):e01044-20. doi: 10.1128/mSystems.01044-20 (PMC7762795; doi:10.1128/mSystems.01044-20)
Supplement: TABLE S1 [file mSystems.01044-20-st001.pdf]

Component per 1 l [mg]	Organic carbon dilution					
	**Full**	**2x**	**5x**	**10x**	**15x**	**20x**
Glucose	500	250	100	50	33	25
Peptone	500	250	100	50	33	25
Yeast extract	500	250	100	50	33	25
Sodium pyruvate	300	150	60	30	20	15
K_2HPO_4	300	300	300	300	300	300
NaCl	1,000	1,000	1,000	1,000	1,000	1,000
