# Supplementary material for: Simultaneous Presence of Bacteriochlorophyll and Xanthorhodopsin Genes in a Freshwater Bacterium
Source: mSystems. 2020 Dec 22;5(6):e01044-20. doi: 10.1128/mSystems.01044-20 (PMC7762795; doi:10.1128/mSystems.01044-20)
Supplement: TABLE S2 [file mSystems.01044-20-st002.pdf]

**Attribute**	**Value**
Total bases	4,382,271
No. of chromosomes	1
Plasmids	3
GC content [%]	65.9
No. of RNAs	60
ncRNAs	3
tRNAs	51
Genes (total)	4,128
- CDS	4,065 (99%)
- hypo. proteins	146 (4%)
- pseudogenes	83 (2%)
PGC length [kbp]	45.4
