# Supplementary material for: Simultaneous Presence of Bacteriochlorophyll and Xanthorhodopsin Genes in a Freshwater Bacterium
Source: mSystems. 2020 Dec 22;5(6):e01044-20. doi: 10.1128/mSystems.01044-20 (PMC7762795; doi:10.1128/mSystems.01044-20)
Supplement: TABLE S3 [file mSystems.01044-20-st003.pdf]

Target	Primer Name	Sequence (5’-3’)
E2E30_16295	pufM-573f	CTCGATCCGCTACGGCAACCTC
	pufM-818r	CACCAGGCCCAGCGATGGATC
E2E30_03390	rpoD-1058f	CTGGGCGGCCAGATGCTGCG
	rpoD-1325r	GCGCCTCGCGCTCGCCCTT
E2E30_05030	PR-319f	GTGATGCGGCTGTCGCGCGAG
	PR-582r	CCCCACGACGCGAAGGTCAGC

Step	Temperature [°C]	Time [s]	Cycles
Initial denaturation	95	600	Hold
Denaturation	95	45	
Annealing	62	45	40
Extending	72	45	
